# Associations between serum 25-hydroxyvitamin D, body mass index and body fat composition among Emirati population: Results from the UAE healthy future study

**DOI:** 10.3389/fendo.2022.954300

**Published:** 2022-10-10

**Authors:** Fatme AlAnouti, Amar Sabri Ahmad, Laila Abdel Wareth, Ayesha Al Dhaheri, Abderrahim Oulhaj, Abdulla Al Junaibi, Abdullah Al Naeemi, Aisha Al Hamiz, Ayesha Al Hosani, Eiman Al Zaabi, Fatima Mezhal, Fatma Al Maskari, Habiba Alsafar, Jamila Yaaqoub, Marina Al Bastaki, Mohammed Al Houqani, Naima Oumeziane, Nirmin F. Juber, Scott Sherman, Syed M. Shah, Teeb Alsharid, Thekra Al Zaabi, Tom Loney, Wael Al Mahmeed, Abdishakur Abdulle, Raghib Ali

**Affiliations:** ^1^ College of Natural and Health Sciences, Zayed University, Abu Dhabi, United Arab Emirates; ^2^ Public Health Research Center, New York University Abu Dhabi, Abu Dhabi, United Arab Emirates; ^3^ Pathology and Laboratory Medicine Institute, Cleveland Clinic Abi Dhabi, Abu Dhabi, United Arab Emirates; ^4^ Department of Nutrition and Health, College of Medicine and Health Sciences, United Arab Emirates University, Abu Dhabi, United Arab Emirates; ^5^ Department of Epidemiology and Public Health, College of Medicine and Health Sciences, Khalifa University of Sciences and Technology, Abu Dhabi, United Arab Emirates; ^6^ Department of Pediatrics, Zayed Military Hospital, Abu Dhabi, United Arab Emirates; ^7^ Department of Cardiology, Zayed Military Hospital, Abu Dhabi, United Arab Emirates; ^8^ Department of Pathology, Sheikh Khalifa Medical City, Zayed Military Hospital, Abu Dhabi, United Arab Emirates; ^9^ Institute of Public Health, College of Medicine and Health Sciences, United Arab University, Abu Dhabi, United Arab Emirates; ^10^ College of Medicine, Zayed Center for Health Sciences, United Arab Emirates University, Abu Dhabi, United Arab Emirates; ^11^ Center for Biotechnology, Khalifa University of Science and Technology, Abu Dhabi, United Arab Emirates; ^12^ Department of Genetics and Molecular Biology, Khalifa University of Science and Technology, Abu Dhabi, United Arab Emirates; ^13^ Department of Biomedical Engineering, Khalifa University of Science and Technology, Zayed Military Hospital, Abu Dhabi, United Arab Emirates; ^14^ Abu Dhabi Police Health Service, Zayed Military Hospital, Abu Dhabi, United Arab Emirates; ^15^ Abu Dhabi Blood Bank Services, Abu Dhabi Health Services Company, SEHA, Abu Dhabi, United Arab Emirates; ^16^ Department of Medicine, College of Medicine and Health Sciences, United Arab University, Abu Dhabi, United Arab Emirates; ^17^ Department of Population Health, New York University School of Medicine, New York, New York, NY, United States; ^18^ College of Medicine, Mohammed Bin Rashid University of Medicine and Health Sciences, Zayed Military Hospital, Dubai, United Arab Emirates; ^19^ Heart and Vascular Institute, Cleveland Clinic Abu Dhabi, Abu Dhabi, United Arab Emirates; ^20^ Medical Research Council, MRC Epidemiology Unit, University of Cambridge, Cambridge, United Kingdom

**Keywords:** body fat, vitamin D, body mass index, serum 25 hydroxyvitamin D, quantile regression

## Abstract

**Introduction:**

Vitamin D deficiency and insufficiency are highly prevalent among several populations across the globe. Numerous studies have shown a significant correlation between body-mass-index (BMI) and Vitamin D status, however, some results differed according to ethnicity. Despite the abundance of sunshine throughout the year, vitamin D deficiency is prominent in the United Arab Emirates (UAE). In this study, we analyzed the UAE Healthy Future Study (UAEHFS) pilot data to investigate the association between serum 25-hydroxyvitamin D (25(OH)D) and % body fat (BF) composition as well as BMI.

**Material and methods:**

Data from a total of 399 Emirati men and women aged ≥ 18 years were analyzed. Serum 25(OH)D and standard measures of weight and height were included in the analyses. Vitamin D deficiency was defined as serum 25(OH)D concentration<20 ng/ml. Multivariate quantile regression models were performed to explore the relationship between serum 25(OH)D levels and % BF composition and BMI correspondingly.

**Results:**

There were 281 (70.4%) males and 118 (29.6%) females included in this study. More than half of the study participants had vitamin D insufficiency (52.4%), and nearly a third had vitamin D deficiency (30.3%); while only 17.3% had optimal levels. A statistically significant negative association between serum 25(OH) D levels and % BF composition was observed at intermediate percentiles while a statistically significant negative association between serum 25(OH)D and BMI was only observed at the median (50th percentile).

**Conclusion:**

The study findings support the association between low serum 25(OH) D levels (low vitamin D status) and high % BF composition and high BMI among adult Emiratis. Further longitudinal data from the prospective UAEHFS could better elucidate the relationship between serum 25(OH) D levels, % BF composition, and BMI in the context of various health outcomes among this population.

## Introduction

Studies around the world regarding the prevalence of vitamin D deficiency (defined by serum 25- hydroxyvitamin D 25(OH)D<50 nmol/l or (<20 ng/mL)), to be approximately 37.3% worldwide (1, 2). There is growing evidence that inadequate vitamin D status, obesity and chronic non-communicable diseases often cluster (3). Evidence from observational studies, demonstrated the high prevalence of vitamin D deficiency in Middle Eastern countries including the United Arab Emirates (UAE) (4–6). Tangible evidence for the role of vitamin D in the regulation of body weight and energy metabolism has been accumulating over the past years (7, 8).

The UAE climate is characterized by a year-round sunny weather. Paradoxically, reports on the vitamin D status among the population have revealed that vitamin D deficiency is highly prevalent among the Emirati UAE nationals across all different age groups but particularly among young adults (5, 9).

Several hypotheses were suggested to explain the notable high prevalence of this public health burden in the UAE. Avoidance of sun due to hot weather, and the perception that sun exposure might be associated with detrimental health effects have been suggested as the main contributing factors. Furthermore, the traditional conservative clothing style contributes to the lack of sun exposure and consequently amplifies the risk of developing vitamin D deficiency (4). Other factors including genetic predisposition and the link of vitamin D deficiency with other chronic diseases among this population had been well documented previously (10, 11).

Two studies have demonstrated a strong association between vitamin D deficiency and obesity, among the Arab adult population of the Gulf Countries (12, 13). The UAE currently ranks fifth in the world in terms of obesity, at a prevalence of 36% (33% males and 39% females) (14, 15). In other words, three in every ten Emirati males and almost 4 out of every 10 females are obese, with an estimated economic burden amounting to $6 billion/year due to obesity-associated complications (16). More than 66% of men and 60% of women in the UAE are currently overweight or obese (an average of 63% or more than double the global average of 30%) (17). This instigates significant health and economic challenges since obesity is an independent risk factor for both metabolic syndrome and some cancers, the major cause of mortality and morbidity in the UAE (14). Since obesity is a multifactorial disease that has several aspects including genetics and environmental conditions, one interesting factor to explore in terms of body energy metabolism is the relation between vitamin D status, body mass index (BMI) and % body fat (% BF) composition in the context of vitamin D deficiency (18).

Serum 25(OH)D levels are frequently used as clinical indicators for the assessment of vitamin D status. With regards to obesity, several indicators including BMI and % BF composition are commonly used (19). In this study, we have aimed to examine the associations of 25(OH)D levels with % BF composition and BMI among a cohort of 399 adult Emiratis.

## Material and methods

### Participants and data acquisition

**Figure 1:**
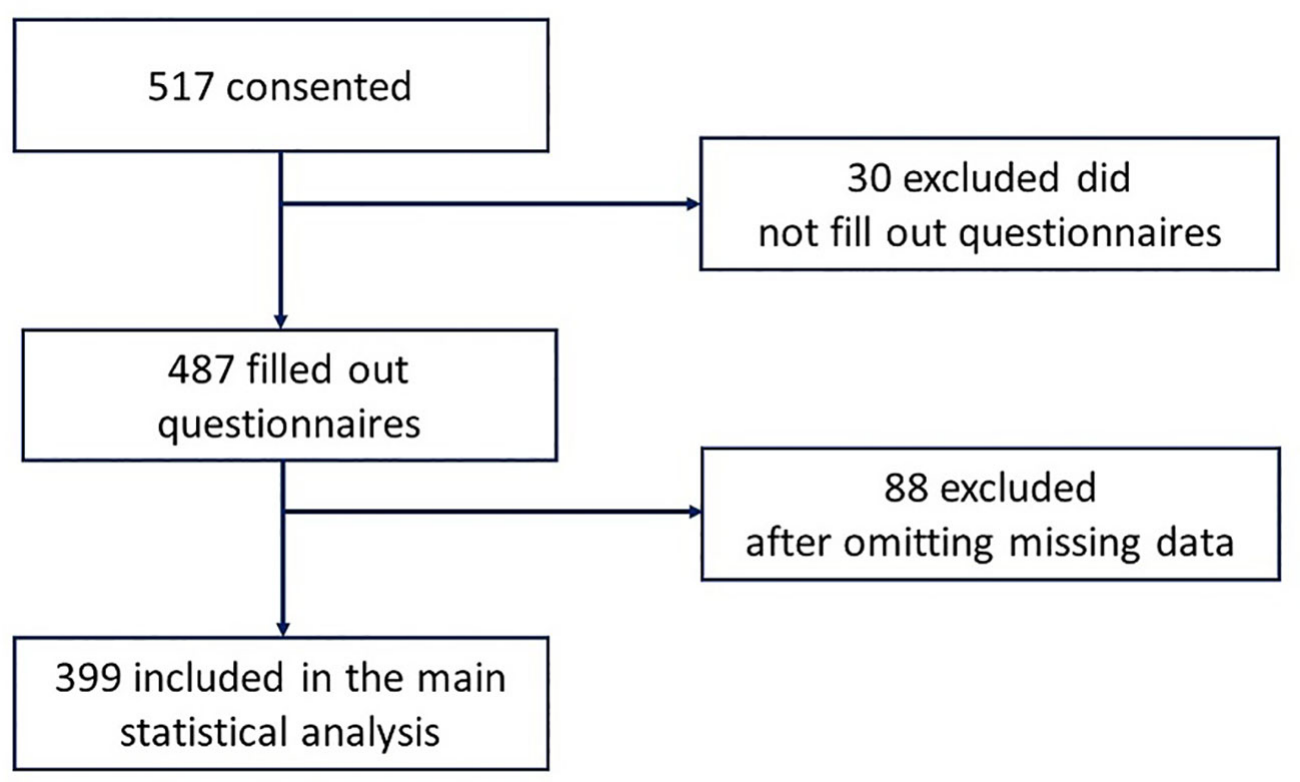
Flow chart of Recruitment of Participants from the UAEHF pilot study.

This study utilized data collected from the electronic records of Emirati participants recruited through the UAE Healthy Future Pilot Study (UAEHFS) (20). Data from a total of 517 Emirati men and women aged over 18 years was analyzed. There was an exclusion for 30 participants because they did not complete the questionnaires and from the remaining 487, a total of 88 cases were further excluded after omitting missing data. The final analysis included data for 399 participants (**Figure 1**). Serum 25-hydroxyvitamin D (25(OH)D) and HBA1c levels along with standard measures of weight, height and % BF composition; were included in the analyses.

### Ethical approval

The UAEHFS was conducted according to the guidelines of the Declaration of Helsinki, and the study protocol was approved by the Research Ethics Committee of Abu Dhabi Health Research and Technology Committee, reference number DOH/HQD/2020/516. All participants read and understood the information leaflet and signed the consent form prior to recruitment.

### Measuring serum 25(OH)D levels

A total of 2 mL of collected blood was used to measure the levels of serum 25(OH)D which were analyzed using chemiluminescence Unicel DXI immunoassay system (Beckman-coulter, USA) with a detection limit 2.0 ng/mL and a total imprecision ≤ 10.0% CV at concentrations greater than 15.0 ng/mL (37.5 nmol/L) (21). Moreover, the total Standard Deviation (SD) was ≤ 1.5 ng/mL (3.8 nmol/L) at concentrations ≤ 15.0 ng/mL. Subjects were categorized according to serum 25(OH) D levels using the following cutoff values: Vitamin D deficient for serum 25(OH) D concentration<20 ng/ml, based on the levels at which bone-related symptoms become apparent, and insufficiency as a serum 25(OH) D of (20–30 ng/ml, determined by the level of vitamin D repletion sufficient for optimum bone (22).

### BMI and % BF composition

BMI and % BF composition were measured using the Tanita MC 780 (Tanita Inc Tokyo, Japan) as previously described (20). Briefly, % BF composition was determined by Bio-impedance and BMI was automatically calculated by the machine as body weight (kg)/height square (meters) with measurements given in kg/m^2^ unit.

### Statistical analysis

Three dimensional scatterplots were presented between all predictors and outcome respectively. Summary statistics were presented by median and interquartile range (IQR) for continuous variables and frequency (percentage) for categorical variables. Quantile regression models were performed to investigate the association between the serum 25(OH) D levels and BF (%) and BMI respectively. The dependent variable was the serum 25(OH) D levels in all fitted multivariate quantile regression models. The 25^th^, 30^th^, 35^th^, 40^th^, 45^th^, 50^th^, 55^th^, 60^th^, 65^th^, 70^th^, 75^th^ percentiles were examined to obtain a representative range of the serum 25(OH) D concentration distribution. In the first eleven fitted multivariate quantile regression models (since we have 11 quantiles), the predictors were age, gender, % BF composition and the interaction term between age and gender (age*gender); while in the second eleven fitted multivariate quantile models, the predictors were age, gender, BMI and the interaction term between age and gender. Statistical analyses were performed using R version 4.1.0 (23).

The sample size of up to 500 participants for the pilot study was determined by the numbers needed to obtain sufficient evidence on the response rate to various recruitment and materials collection strategies and to assist in sample size calculations for the main study.

## Results

Out of 517 participants who consented to participate in the UAEHF pilot study, 487 (94.2%) had complete questionnaire data (18). Data from 399 (77.2%) participants were included in this statistical analysis after omitting missing values (**Figure 1**). The majority of participants were classified as having vitamin D insufficiency 209 (52.4%) with females 67 (32.1%) and males 142 (67.9%). Furthermore, there were 121 (30.3%) subjects with vitamin D deficiency with 24 (19.8%) females and 97 (80.2%) males. Moreover, 69 (17.3%) of the participants were vitamin D sufficient with 27 (39.1%) females and 42 (60.9%) males. A statistically significant difference was observed in serum 25(OH)D levels between females and males (Chi square 0.0103).

**Table 1** presents summary statistics (median and interquartile range) for participants’ characteristics and clinical investigations of the age, serum 25(OH) D concentrations, % BF, BMI, and HBA1c variables. The median age of the UAEHFS pilot data participants was 31 years (Interquartile Range: 24 – 38) with 281 (70.4%) males and (29.6%) females. Females had lower median serum 25(OH) D concentrations compared to males, median (IQR) of 18.7(13.3, 27.2) and 19.8 (16.5, 25.3) respectively; which is below the sufficiency levels. No statistically significant difference was observed in serum 25(OH) D levels between females and males p-value=0.120. Furthermore, females had lower BMI median than males, median (IQR) of 27 (22.9, 33) and 28.1 (24.2, 31.7), however, the difference was not statistically significant Wilcoxon p-value = 0.452. No statistically significant difference in the HBA1c measurement was observed between females and males, Wilcoxon p-value = 0.920 (**Table 1**). Moreover, out of the 399 participants, there were 28 (7%) and 83 (21%) participants diabetic and hypertensive respectively

**Table 1:** Median (interquartile range) of the variables included in the statistical analysis as well as Wilcoxon rank sum test p-value.

Variable	All (n = 399)	Females (n = 118)	Males (n = 281)	P-value
Age (year)	30 (23, 38)	30 (24, 37)	31 (24, 38)	0.461
25(OH)D (ng\mL)	19.5 (15.5, 25.6)	18.7 (13.3, 27.2)	19.8 (16.5, 25.3)	0.120
Body Fat (%)	27.5 (22.3, 33.4)	35.0 (29.8, 40.7)	25.7 (21, 29.7)	0.876*
BMI (kg/m2)	27.8 (23.8, 32)	27.0 (22.9, 33)	28.1 (24.2, 31.7)	0.452
HBA1c (mmol/mol)	5.4 (5.1, 5.7)	5.4 (5.1, 5.7)	5.4 (5.1, 5.7)	0.920

*P-value from a multivariate quantile regression model with age, gender and age*gender as predictors and % BF composition as an outcome. Wilcoxon rank sum test was not performed as this is an age and gender specific test for % BF.

**Figure 2:**
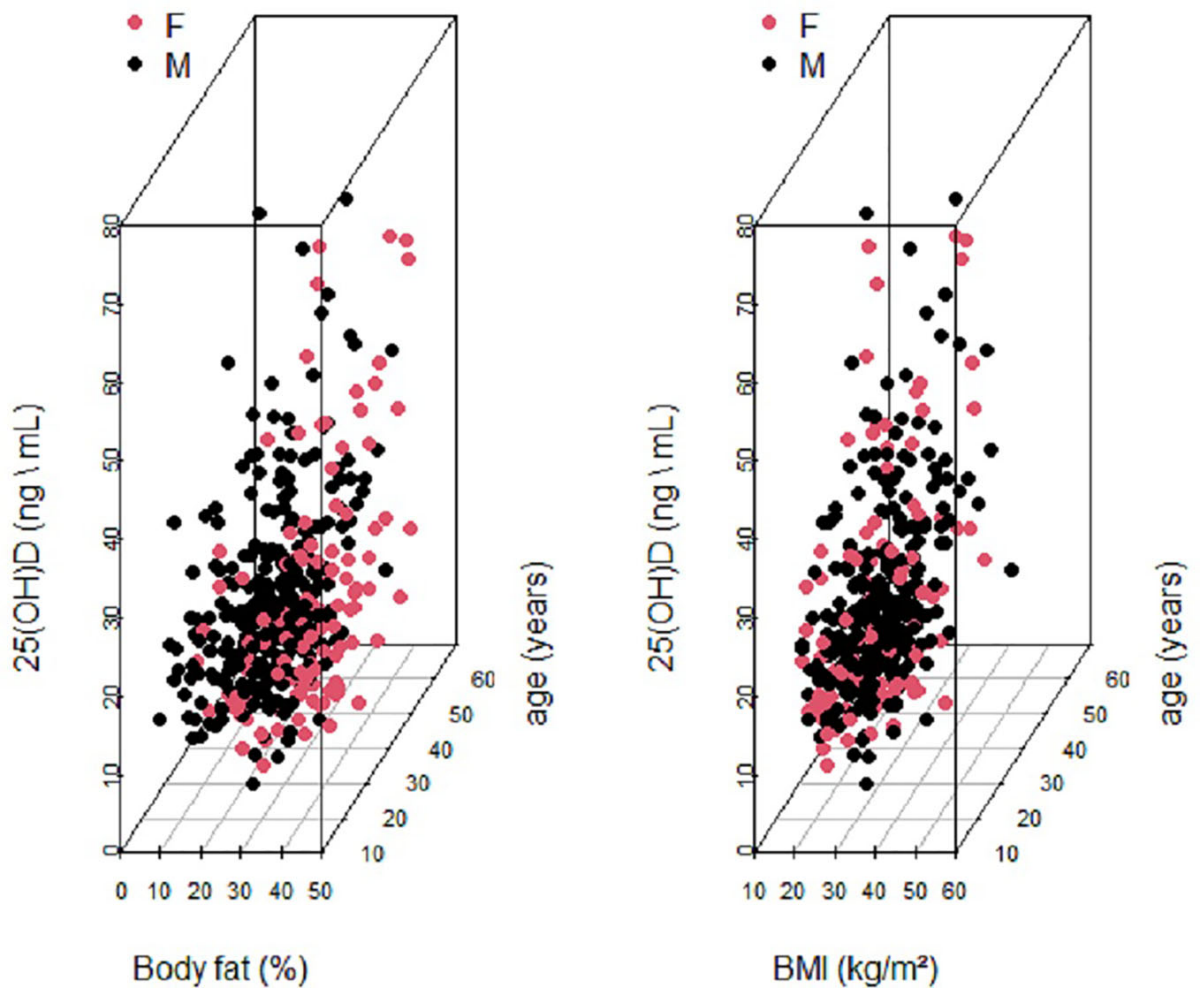
Two 3-Dimensional Scatterplots of Serum 25 (OH)D (ng\ml) versus age and BF (%) and age and BMI respectively. Data is labeled by sex: male (blackdots) and female (red dots).

**Figure 2** shows two 3-dimensional scatterplots of serum 25(OH) D versus age and % BF composition and age and BMI respectively. The data was plotted for both males and females to enable us to explore relationships between the four variables at the same time. A clear separation by sex was observed for serum 25(OH) D versus % BF and age (**Figure 2** left graph). No separation by sex was observed for serum 25(OH) D versus BMI and age (**Figure 2** right graph).

**Figure 3:**
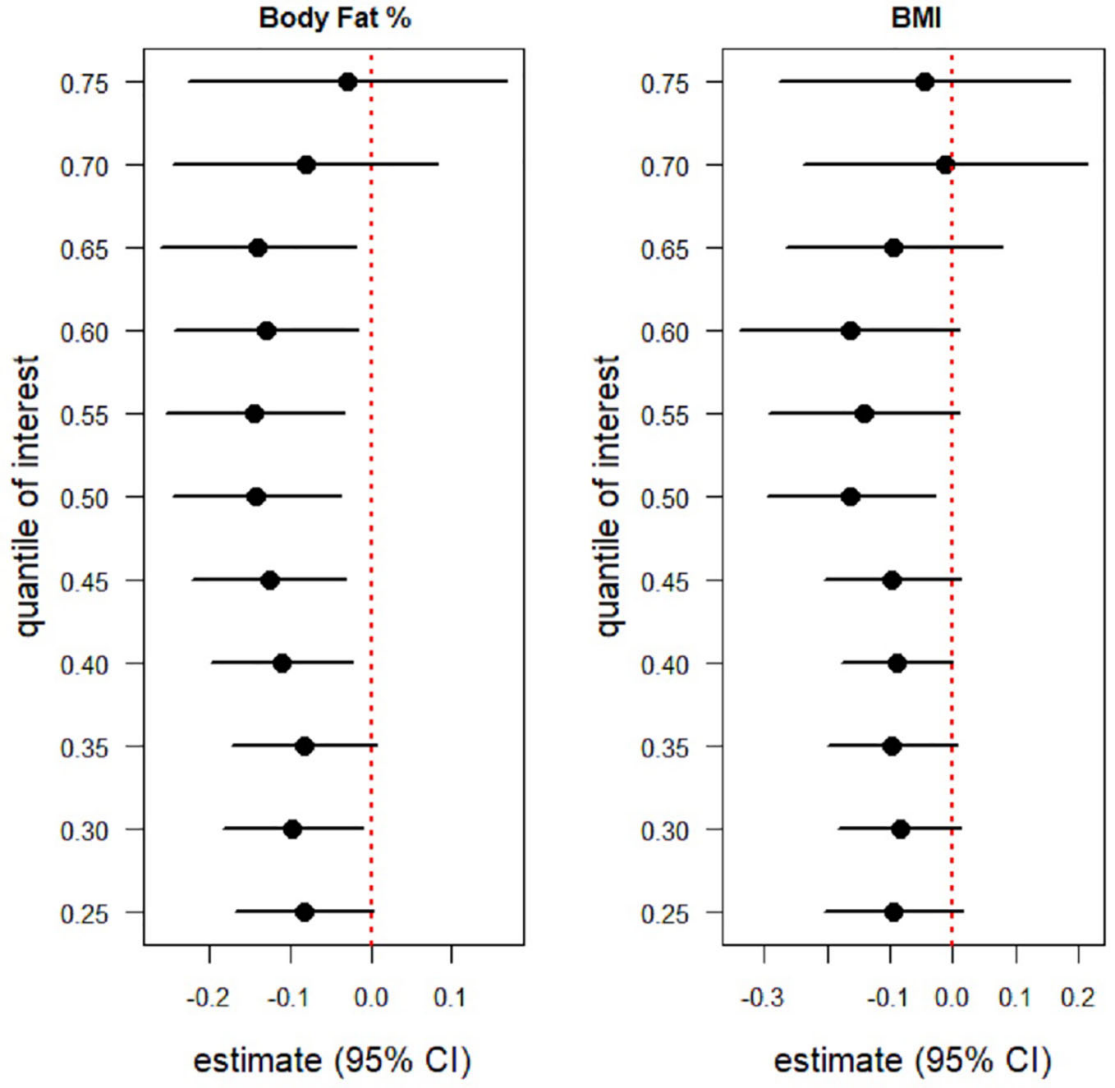
Caterpillar plots of the BMI and BF (%) effects on serum 25(OH)D levels from two multivariate quantile regression model for the 25^th^, 30^th^,...,75^th^ percentile .Each black dot is the slope coeficiant for the quantile indicated on the y axis. If 95% confidence intervals are below zero (red dotted line) indicating a statistically significant negative association, if 95% confidnece intervals cross the red dotted line then there is no statistically significant effect. Body Fat (%) is negatively associated with 25(OH)D level at the 25^th^, 30^th^,..., 75^th^ percentile. statistically significant negative associations were observed between Body Fat (%) and 25(OH)D level at the intermediation percentile (40^th^,...,65^th^). BMI was also negatively associated with 25(OH)D levels at 25^th^, 30^th^,...,, 75^th^ percentile. However, a statistically significant negative association between BMI and 25(OH)D levels was only observed at the intermediate percentile (40^th^,..., 65^th^).

**Figure 3** shows caterpillar plots of the estimated relationship between 25(OH) D levels and BMI and % BF from multivariate quantile regression models for the 25^th^, 75^th^ percentiles levels. This figure also illustrates that % BF composition was negatively associated with serum 25(OH) D concentrations at the 25^th^, 30^th^, 75^th^ percentile. Statistically significant negative associations were observed between serum 25(OH)D level and BMI at the intermediate percentiles (**Figure 3**). The estimated effect (95% CI) of the 40^th^, 45^th^, 50^th^, 55^th^, 60^th^, 65^th^ percentile was -0.110 (-0.198, -0.022), -0.127 (-0.222, -0.032), -0.142 (-0.245, -0.039), -0.145 (-0.255, -0.034), -0.130 (-0.244, -0.017), -0.140 (-0.261, -0.019), correspondingly (**Supplementary Table 1**).

Similarly, BMI was also negatively associated with intermediate percentiles. However, a statistically significant negative association between serum 25(OH)D level and BMI was only observed at the 50^th^ percentile (median), estimated effect (95% CI) of -0.163 (-0.296, -0.029). This indicates a weaker relation in comparison with % BF (**Supplementary Table S1**).

**Supplementary Figure S1** shows the difference in 25(OH)D levels between males and females in the fitted quantile regression models. In general, males have higher serum 25(OH)D levels as compared to females, however it was only statistically significant at the 35^th^, 55^th^, 60^th^, 65^th^ and 70^th^ percentiles for both models with BMI and %BF composition, respectively. In addition, there was a significant difference in 25(OH)D levels between males and females at the median level in the multivariate quantile regression model with age, sex and BMI as predictors.

## Discussion

Hypovitaminosis and obesity are highly prevalent in the UAE (14). This study presents interesting findings regarding the association between vitamin D status, BMI, and % BF composition among a cohort of Middle Eastern adult nationals from the UAE. Our data revealed that vitamin D deficiency was common among adult Emiratis of both genders (median serum 25(OH)D level of 19.48 ng/ml) in agreement with results demonstrated by other researchers (4, 5, 24). This is also consistent with a previous retrospective study on Emirati nationals which demonstrated that 67.1% of men and 73.5% of women were suffering from vitamin D deficiency with the lowest levels being reported among young adult Emiratis (5). Moreover, an investigation of the vitamin D status in a sample of 208 young adults in Abu Dhabi, similarly revealed that vitamin D deficiency was highly prevalent among both genders but more significantly among females (mean serum 25(OH)D of 20.9 ± 14.9 nmol/L for females versus 27.3 ± 15.7 nmol/L for males) and attributed reasons to high levels of sun avoidance (4). Furthermore, another retrospective study of the UAE population reported the mean value for serum 25(OH)D to be 48.89 nmol/L with 82% of 60,979 patients presenting with hypovitaminosis, of which 26% of females and 18% of males had severe deficiency (25). Among the examined participants, 30,640 (50%) were UAE nationals. Almost 86.1% of UAE nationals had serum levels of <75 nmol/L of 25(OH)D, hence demonstrating the serious magnitude of this public health burden among both male and female Emiratis (25).

In the present study, we aimed to examine the associations of 25(OH)D levels with % BF composition and BMI in a cohort of adult Emiratis. In our investigation, quantile regression models were used with different percentiles instead of categorizing vitamin D data to demonstrate point estimation across the vitamin-D distribution beyond the median. This is because we were interested in investigating the association with % BF composition and with BMI at different quantiles. Furthermore, a quantile regression would also allow the investigation of these associations under heteroscedasticity and non-normality assumptions. The results from **Figure 3** revealed a negative association for serum 25(OH)D levels with both % BF composition and BMI, although more remarkably over more percentiles with % BF composition as compared to BMI. This is in line with recent systematic reviews and meta-analysis confirming the negative association between low serum 25(OH)D levels and high % BF composition and high BMI in the context of obesity among adults (18, 26, 27).

Few studies from the UAE had examined the relationship of vitamin D status with BMI but not with % BF composition (4–6). Although a negative association was like wise reported with BMI, only one study did not find any correlation between serum 25(OH)D levels and BMI (4). Previous researchers highlighted the major limitation of the BMI when it comes to differentiating between muscle and fat (28). Moreover, others demonstrated that the use of BMI was not precise to predict adult obesity as it is known to systematically underestimate or overestimate adiposity in certain ethnic groups because of its dependency on the height measurement, as well as lean body and bone mass (29).

The Several factors including the drastic life style changes, low physical activity, less incidental exposure to sunlight and unhealth dietary practices had led to the remarkable prevalence of obesity and metabolic diseases among the population of the Middle Eastern and North African countries (30).

In this study, lower mean serum 25(OH)D levels were correlated with higher % BF composition. Similar studies by Snijder et al. and Muscogiuri et al. found an inverse association between total BF and serum 25(OH)D concentrations in both men and women (31, 32). Additional research among Caucasians and Africans have yielded concordant results (33). The mechanism by which BF results in lower vitamin D levels is related to volumetric dilution and diminished vitamin D synthesis in the adipose tissue and liver, however this is yet to be fully elucidated. One of the highly likely mechanisms is the sequestration of vitamin D by adipose tissue and depletion of body from vitamin D by the obesity associated inflammation (24–27). Another contributing factor could be due to the enhanced action of 24-hydroxylase enzyme, which is found in adipose tissue. Likewise, a study in the Netherlands on 2158 older adults showed that vitamin D deficient participants (25(OH)D < 50 nmol/L) had a higher BF % (β = 1.29, 95% CI: 0.55, 2.04) (34).

Our results showed gender differences in terms of % BF composition and BMI when interpreting vitamin D deficiency results. These results are in agreement with the findings reported from a cross-sectional study by Kremer et al. which found that vitamin D insufficiency was associated with increased BF in young women and another observational prospective cohort among adults revealing lower vitamin D concentrations in females than males by Muscogiuri et al. (32, 35). These findings could be interpreted by the fact that women generally have higher BF than males (26).

The relationship between % BF composition and vitamin D could depend on ethnicity. Data from a study with 1,697 recruited Korean adults aiming to examine the relation between serum 25(OH)D as an indicator of vitamin D deficiency [25(OH)D <20 ng/mL] and BMI and BF revealed that serum 25(OH)D levels showed significant negative correlation with BF but not BMI (36).

This was confirmed by a systematic review that depicted a difference between Asians and Caucasians in terms of BMI and % BF composition (37). Similarly, previous research among Caucasians and Africans, has yielded concordant results in terms of BMI and vitamin D status (33). Nonetheless, several modifiable risk factors of low vitamin D status were revealed by a study among middle-aged women and men in the US and included inadequate vitamin D intake, a BMI or >30 kg/m^2^, physical inactivity as well as low intake of dairy and calcium supplements (38). In the current study, a clear separation by sex was observed for serum 25(OH)D versus BF (%) and age. A systematic review by Mithal et al. on global vitamin D deficiency and its determinants revealed that risk factors for deficiency comprise female gender, lower latitude, season variations, dark skin pigmentation, sunlight exposure and its associated determining factors such as clothing, dietary practices as well as national policies of vitamin D fortification (39).

According to current statistics, 85% of the UAE residents suffer from Vitamin D deficiency. The UAE is known to enjoy year-round sunny weather. Vitamin D deficiency and the rapid increase in the prevalence of obesity are both considered important public health issues contributing significantly to modern healthcare costs, morbidity, and mortality. The strong association between these two modern health issues is not new. A series of evidence supports the fact that obesity might be driving low serum 25(OH)D concentration due to decreased bioavailability of vitamin D through sequestration in body fat compartments. However, recent studies have opened up a new dimension regarding the role of vitamin D in the regulation of body weight. Vitamin D supplementation effects on body weight remain a controversy. A review article by Golzarand et al. concluded that supplementation with vitamin D had no impact on body fat percentage (40). While conversely, in a study by Salehpour et al. results of a 12-week double-blind randomized clinical trial of vitamin D supplementation, showed that increasing vitamin D levels by supplementation resulted in BF mas lessening (41). Similarly, a study among collegiate athletes concluded that Vitamin D supplementation can help regulate and maintain the athletes’ % BF in times of low sports activity (42). Interestingly, a recent systematic review and meta-analysis of randomized controlled trials found that supplementation during pregnancy or infancy could be associated with less likelihood of adiposity during childhood (43). These findings are promising and pave the way for larger-scale studies on the impact of vitamin D adequacy and supplementation on body composition.

The results of the present study underscore important implications for vitamin D research. First, the data confirm the negative correlation between % BF composition and vitamin D status among a cohort sample of Emirati adults. Second, the data calls for a need to screen for deficiency in individuals with high % BF composition regardless if their BMI was low. Although BMI is significantly correlated with total fat mass, it still cannot reliably predict fat mass in individuals. BMI has limited value when measuring body composition. Both lean mass and fat mass correlate very well with BMI. Despite this, BMI can only be used to follow short-term changes in body composition as changes in the BMI standard deviation score in the short term can be attributed primarily to changes in fatness (44). Further, this study underscores the importance of utilizing different obesity indicators including % BF to assess for abdominal obesity which significantly contributes to metabolic syndrome. The use of multiple obesity indices is important to obtain a comprehensive picture of this burden. Abdominal obesity has been associated with visceral fat mass, higher blood pressure, and cardiovascular risk factor clustering, and should be measured along with BMI. With regards to vitamin D, evidence shows that vitamin D deficiency is associated with risk factors for metabolic syndrome and showed the positive effects of vitamin D supplementation in improving outcomes in people diagnosed with metabolic syndrome (45, 46). Therefore, there is an urgent need for a standardized definition of obesity in clinical practice as well as in relevant epidemiological settings.

## Strengths and limitations

This study examined the association between vitamin D data and BF% and BMI among a sample of nationals within the UAE. We used an objective measure of serum 25(OH)D for our main exposure to minimize misclassification of our participants’ vitamin D levels. We used two indicators of obesity, namely BMI and % BF composition, to allow us to compare their predictive performance for vitamin D deficiency. Our results provided implications that % BF could be a better predictor for vitamin D deficiency compared to BMI. BMI as an indicator of % BF is commonly used in the epidemiological population study because it is easy to measure, however, previous study has found that the association between BMI and % BF composition was not strong, especially among those with BMI <27 kg/m2 (47). Finally, we used multivariate quantile regression analyses to control for confounding variables and enable us to explore the influence of age, gender, BMI and % BF composition at different quantiles of the vitamin D distribution. Despite the important implications of our study, there are some limitations that are worth noting. The optimal concentration of serum 25(OH)D for overall health remains very controversial and using different cut offs might slightly change results. The dietary intake of vitamin D along with any use of supplements and sun exposure were not recorded but could have affected the vitamin D status significantly. Future larger studies that examine all determinants of vitamin D status are warranted before conclusive results are confirmed. The final limitation comes from omitting missing values on vitamin D data.

## Conclusion

The result of this study raises a main concern for population health, which requires further studies. The majority of the UAEHF pilot study participants were vitamin D deficient. The results also support the association between low serum 25(OH) D on one side and high % BF composition and high BMI on the other in adult Emiratis. The association with % BF composition could be more informative and accurate; however, further additional studies are needed to clarify this observed negative correlation.

## Data availability statement

The raw data supporting the conclusions of this article will be made available by the authors, without undue reservation.

## Ethics statement

The studies involving human participants were reviewed and approved by The UAEHFS was conducted according to the guidelines of the Declaration of Helsinki, and the study protocol was approved by the Research Ethics Committee of Abu Dhabi Health Research and Technology Committee, reference number DOH/HQD/2020/516. All participants read and understood the information leaflet and signed the consent form prior to recruitment. The patients/participants provided their written informed consent to participate in this study.

## Author contributions

FA: Conceptualization, data interpretation, writing of original draft. ASA, AA, RA: Conceptualization, Project administration, writing and editing. ASA: Conceptualization, Data analysis and data interpretation. All authors have contributed to draft writing and final revision. All authors read and agreed to the published version of the manuscript.

## Funding

This publication is based upon works supported by Tamkeen under Research Institute Grant No. G1206.

## Conflict of interest

MB and NO were employed by Abu Dhabi Health Services Company, SEHA.

The remaining authors declare that the research was conducted in the absence of any commercial or financial relationships that could be construed as a potential conflict of interest.

## Publisher’s note

All claims expressed in this article are solely those of the authors and do not necessarily represent those of their affiliated organizations, or those of the publisher, the editors and the reviewers. Any product that may be evaluated in this article, or claim that may be made by its manufacturer, is not guaranteed or endorsed by the publisher.

## References

[1] Hilger J Friedel A Herr R Rausch T Roos F Wahl DA. A systematic review of vitamin d status in populations worldwide. Br J Nutr (2014) 111(1):23–45. doi: 10.1017/S0007114513001840 23930771

[2] Lips P Cashman KD Lamberg-Allardt C Bischoff-Ferrari HA Obermayer-Pietsch B Bianchi ML. Current vitamin d status in European and middle East countries and strategies to prevent vitamin d deficiency: a position statement of the European calcified tissue society. Eur J Endocrinol (2019) 180(4):P23–54. doi: 10.1530/EJE-18-0736 30721133

[3] Soares M Chan She Ping-Delfos W Ghanbari M. Calcium and vitamin d for obesity: a review of randomized controlled trials. Eur J Clin Nutr (2011) 65(9):994–1004. doi: 10.1038/ejcn.2011.106 21731038

[4] Al Anouti F Thomas J Abdel-Wareth L Rajah J Grant WB Haq A. Vitamin d deficiency and sun avoidance among university students at Abu Dhabi, united Arab Emirates. Derm-Endocrinol (2011) 3(4):235–9. doi: 10.4161/derm.3.4.16881 PMC3256339 22259650

[5] Buckley AJ Barakat MT Holick MF Lessan N. Parameters of bone and cardiovascular health related to 25-hydroxyvitamin d status in emirati nationals attending primary care and diabetes services: a retrospective cohort study. Sci Rep (2019) 9(1):1–8. doi: 10.1038/s41598-019-40523-8 30846793 PMC6405844

[6] Nimri LF. Vitamin d status of female UAE college students and associated risk factors. J Public Health (2018) 40(3):e284–e90. doi: 10.1093/pubmed/fdy009 29385507

[7] Lupton JR Faridi KF Martin SS Sharma S Kulkarni K Jones SR. Deficient serum 25-hydroxyvitamin d is associated with an atherogenic lipid profile: The very Large database of lipids (VLDL-3) study. J Clin Lipidol (2016) 10(1):72–81.e1. doi: 10.1016/j.jacl.2015.09.006 26892123 PMC4762185

[8] Mitri J Dawson-Hughes B Hu FB Pittas AG. Effects of vitamin d and calcium supplementation on pancreatic β cell function, insulin sensitivity, and glycemia in adults at high risk of diabetes: the calcium and vitamin d for diabetes mellitus (CaDDM) randomized controlled trial. Am J Clin Nutr (2011) 94(2):486–94. doi: 10.3945/ajcn.111.011684 PMC3142723 21715514

[9] Buckley AJ Hannoun Z Lessan N Holick MF Barakat MT. Environmental determinants of previtamin d synthesis in the united Arab Emirates. Derm-Endocrinol (2017) 9(1):e1267079. doi: 10.1080/19381980.2016.1267079 PMC5893264 29657665

[10] Al Anouti F Chehadeh SEH Osman E ElGhazali G Al Safar H. Investigating the association of vitamin d metabolism genes CYP2R1, CYP24A1 and CYP27B1 with vitamin d status in young adult emiratis. J Food Nutr Res (2017) 5(1):15–21. doi: 10.12691/jfnr-5-1-3

[11] Osman E Al Anouti F Haq A Mirgani R Al Safar H. Frequency of rs731236 (Taql), rs2228570 (Fok1) of vitamin-d receptor (VDR) gene in emirati healthy population. Meta Gene (2015) 6:49–52. doi: 10.1016/j.mgene.2015.09.001 26504744 PMC4576359

[12] Al-Mahroos F Al-Roomi K. Overweight and obesity in the Arabian peninsula: an overview. J R Soc Promot Health (1999) 119(4):251–3. doi: 10.1177/146642409911900410 10673848

[13] Samara A Andersen PT Aro AR. Health promotion and obesity in the Arab gulf states: challenges and good practices. J Obes (2019) 2019. doi: 10.1155/2019/4756260 PMC6590587 31281673

[14] Health authority Abu dhabi. health statistics (2016). Available at: https://www.doh.gov.ae/en/resources/opendata.

[15] World Health Organization. Obesity and overweight (2021). Available at: https://www.who.int/news-room/fact-sheets/detail/obesity-and-overweight.

[16] Mckinsey Global Institute. The global obesity threat (2015). Available at: https://www.mckinsey.com/mgi/overview/in-the-news/the-global-obesity-threat.

[17] Ng M Fleming T Robinson M Thomson B Graetz N Margono C. Global, regional, and national prevalence of overweight and obesity in children and adults during 1980–2013: a systematic analysis for the global burden of disease study 2013. Lancet (9945) 2014:766–81:384. doi: 10.1016/s0140-6736(14)60460-8"10.1016/S0140-6736(14)60460-8 PMC4624264 24880830

[18] Hajhashemy Z Shahdadian F Ziaei R Saneei P. Serum vitamin d levels in relation to abdominal obesity: A systematic review and dose–response meta-analysis of epidemiologic studies. Obes Rev (2021) 22(2):e13134. doi: 10.1111/obr.13134 32881271

[19] Centers for disease control and prevention. defining adult overweight and obesity (2020). Available at: https://www.cdc.gov/obesity/basics/adult-defining.html.

[20] Abdulle A Alnaeemi A Aljunaibi A Al Ali A Al Saedi K Al Zaabi E. The UAE healthy future study: a pilot for a prospective cohort study of 20,000 united Arab Emirates nationals. BMC Public Health (2018) 18(1):101. doi: 10.1186/s12889-017-5012-2 29304844 PMC5755402

[21] Topçuoğlu C Sezer S Yılmaz FM Kösem A Ercan M Turhan T. Evaluation of the analytical performance of the beckman coulter unicel DXI 800 access total 25 (OH) vitamin d immunoassay. LaboratoriumsMedizin (2018) 42(5):205–11. doi: 10.1515/labmed-2018-0068

[22] Holick MF Binkley NC Bischoff-Ferrari HA Gordon CM Hanley DA Heaney RP. Evaluation, treatment, and prevention of vitamin d deficiency: an endocrine society clinical practice guideline. J Clin Endocrinol Metab (2011) 96(7):1911–30. doi: 10.1210/jc.2011-0385 21646368

[23] R Core Team. R: A language and environment for statistical computing. Vienna, Austria: R Foundation for Statistical Computing (2021). Available at: https://www.R-project.org/.

[24] Chakhtoura M Rahme M Chamoun N Fuleihan GE-H. Vitamin d in the middle East and north Africa. Bone Rep (2018) 8:135–46. doi: 10.1016/j.bonr.2018.03.004 PMC6020111 29955632

[25] Haq A Svobodová J Imran S Stanford C Razzaque MS. Vitamin d deficiency: A single centre analysis of patients from 136 countries. J Steroid Biochem Mol Biol (2016) 164:209–13. doi: 10.1016/j.jsbmb.2016.02.007 26877203

[26] Karampela I Sakelliou A Vallianou N Christodoulatos G-S Magkos F Dalamaga M. Vitamin d and obesity: Current evidence and controversies. Curr Obes Rep (2021) 10(2):162–80. doi: 10.1007/s13679-021-00433-1 33792853

[27] Rafiq S Jeppesen PB. Body mass index, Vitamin D, And type 2 diabetes: A systematic review and meta-analysis. Nutrients (2018) 10(9):1182. doi: 10.3390/nu10091182 PMC6164132 30154381

[28] Rothman KJ. BMI-related errors in the measurement of obesity. Int J Obes (2008) 32(3):S56–S9. doi: 10.1038/ijo.2008.87 18695655

[29] Weng SF Redsell SA Nathan D Swift JA Yang M Glazebrook C. Estimating overweight risk in childhood from predictors during infancy. Pediatrics (2013) 132(2):e414–21. doi: 10.1542/peds.2012-3858 23858427

[30] Nasreddine L Ayoub JJ Al Jawaldeh A Mediterranean WHOROftE. Review of the nutrition situation in the Eastern Mediterranean region. East Mediterr Health J (2018) 24(01):77–91. doi: 10.26719/2018.24.1.77 29658624

[31] Snijder MB van Dam RM Visser M Deeg DJH Dekker JM Bouter LM. Adiposity in relation to vitamin d status and parathyroid hormone levels: A population-based study in older men and women. J Clin Endocr (2005) 90(7):4119–23. doi: 10.1210/jc.2005-0216 15855256

[32] Muscogiuri G Barrea L Somma CD Laudisio D Salzano C Pugliese G. Sex differences of vitamin d status across BMI classes: An observational prospective cohort study. Nutrients (2019) 11(12):3034. doi: 10.3390/nu11123034 PMC6950363 31842281

[33] Rajakumar K de las Heras J Chen TC Lee S Holick MF Arslanian SA. Vitamin d status, adiposity, and lipids in black American and Caucasian children. J Clin Endocrinol Metab (2011) 96(5):1560–7. doi: 10.1210/jc.2010-2388 PMC3085205 21367931

[34] Vitezova A Muka T Zillikens MC Voortman T Uitterlinden AG Hofman A. Vitamin d and body composition in the elderly. Clin Nutr (2017) 36(2):585–92. doi: 10.1016/j.clnu.2016.04.017 27346177

[35] Kremer R Campbell PP Reinhardt T Gilsanz V. Vitamin d status and its relationship to body fat, final height, and peak bone mass in young women. J Clin Endocr (2009) 94(1):67–73. doi: 10.1210/jc.2008-1575 18984659 PMC2630864

[36] Han SS Kim M Lee SM Lee JP Kim S Joo KW. Association between body fat and vitamin d status in Korean adults. Asia Pac J Clin Nutr (2014) 23(1):65–75. doi: 10.6133/apjcn.2014.23.1.10 24561974

[37] Deurenberg P Deurenberg-Yap M Guricci S. Asians are different from caucasians and from each other in their body mass index/body fat per cent relationship. Obes Rev (2002) 3(3):141–6. doi: 10.1046/j.1467-789X.2002.00065.x 12164465

[38] Brock K Huang WY Fraser DR Ke L Tseng M Stolzenberg-Solomon R. Low vitamin d status is associated with physical inactivity, obesity and low vitamin d intake in a large US sample of healthy middle-aged men and women. J Steroid Biochem Mol Biol (2010) 121(1):462–6. doi: 10.1016/j.jsbmb.2010.03.091 PMC2906665 20399270

[39] Mithal A Wahl DA Bonjour JP Burckhardt P Dawson-Hughes B Eisman JA. Global vitamin d status and determinants of hypovitaminosis d. Osteoporos Int (2009) 20(11):1807–20. doi: 10.1007/s00198-009-0954-6 19543765

[40] Golzarand M Hollis BW Mirmiran P Wagner CL Shab-Bidar S. Vitamin d supplementation and body fat mass: a systematic review and meta-analysis. Eur J Clin Nutr (2018) 72(10):1345–57. doi: 10.1038/s41430-018-0132-z 29563638

[41] Salehpour A Hosseinpanah F Shidfar F Vafa M Razaghi M Dehghani S. A 12-week double-blind randomized clinical trial of vitamin D3supplementation on body fat mass in healthy overweight and obese women. Nutr J (2012) 11(1):1–8. doi: 10.1186/1475-2891-11-78 22998754 PMC3514135

[42] Kawashima I Tsukahara T Kawai R Mizuno T Ishizuka S Hiraiwa H. The impact of vitamin d supplementation on body fat mass in elite male collegiate athletes. Nutr Metab (2021) 18(1):51. doi: 10.1186/s12986-021-00578-9 PMC8138511 34020679

[43] Ma K Wei SQ Bi WG Weiler HA Wen SW. Effect of vitamin d supplementation in early life on children’s growth and body composition: A systematic review and meta-analysis of randomized controlled trials. Nutrients (2021) 13(2):524. doi: 10.3390/nu13020524 33562750 PMC7914476

[44] Davies P. Body composition assessment. Arch Dis Childh (1993) 69(3):337. doi: 10.1136/adc.69.3.337 8215541 PMC1029514

[45] Prasad P Kochhar A. Interplay of vitamin d and metabolic syndrome: A review. Diabetes Metab Syndr.: Clin Res Rev (2016) 10(2):105–12. doi: 10.1016/j.dsx.2015.02.014 25813139

[46] Melguizo-Rodríguez L Costela-Ruiz VJ García-Recio E De Luna-Bertos E Ruiz C Illescas-Montes R. Role of vitamin d in the metabolic syndrome. Nutrients (2021) 13(3):830. doi: 10.3390/nu13030830 33802330 PMC7999005

[47] Meeuwsen S Horgan G Elia M. The relationship between BMI and percent body fat, measured by bioelectrical impedance, in a large adult sample is curvilinear and influenced by age and sex. Clin Nutr (2010) 29(5):560–6. doi: 10.1016/j.clnu.2009.12.011 20359792

