# Supplementary figures and images for: Associations between serum 25-hydroxyvitamin D, body mass index and body fat composition among Emirati population: Results from the UAE healthy future study
Source: Front Endocrinol (Lausanne). 2022 Oct 10;13:954300. doi: 10.3389/fendo.2022.954300 (PMC9589411; doi:10.3389/fendo.2022.954300)

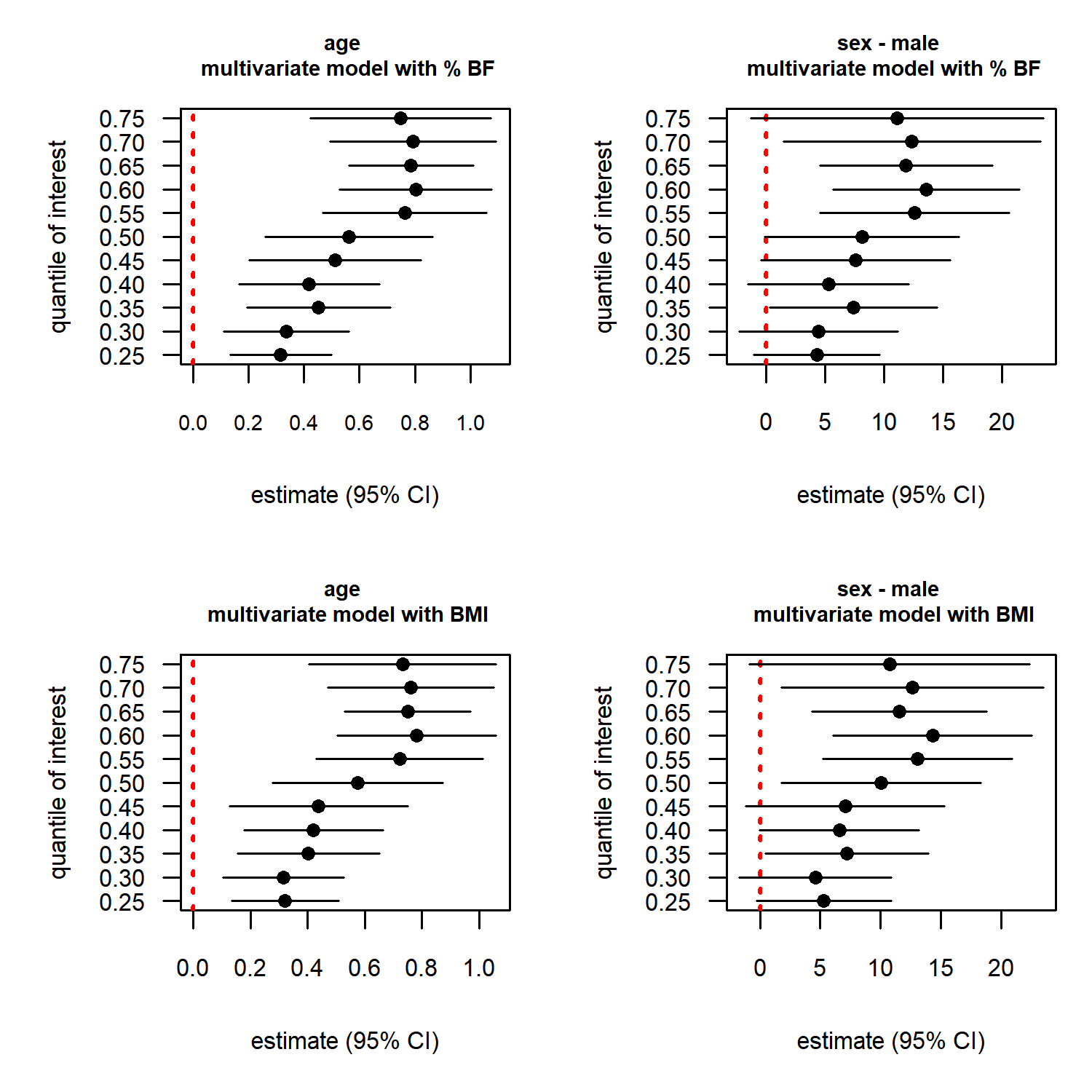

Supplement: Supplementary file 1 [file Image_1.tiff]
