# Supplementary material for: Associations between serum 25-hydroxyvitamin D, body mass index and body fat composition among Emirati population: Results from the UAE healthy future study
Source: Front Endocrinol (Lausanne). 2022 Oct 10;13:954300. doi: 10.3389/fendo.2022.954300 (PMC9589411; doi:10.3389/fendo.2022.954300)
Supplement: Supplementary file 2 [file Table_1.docx]

| **Supplementary Table 1**  The estimated effect (95% confidence interval) of BMI and BF (%) at Different Percentiles   \| percentile \| estimate \| 95% lower \| 95% upper \| Variable \| \| --- \| --- \| --- \| --- \| --- \| \| 0.25 \| -0.094 \| -0.204 \| 0.017 \| BMI \| \|  \| -0.083 \| -0.168 \| 0.003 \| % BF \| \| 0.30 \| -0.084 \| -0.183 \| 0.014 \| BMI \| \|  \| -0.098 \| -0.184 \| -0.011 \| % BF \| \| 0.35 \| -0.096 \| -0.199 \| 0.006 \| BMI \| \|  \| -0.083 \| -0.174 \| 0.007 \| % BF \| \| 0.40 \| -0.088 \| -0.176 \| 0.001 \| BMI \| \|  \| -0.110 \| -0.198 \| -0.022 \| % BF \| \| 0.45 \| -0.097 \| -0.205 \| 0.012 \| BMI \| \|  \| -0.127 \| -0.222 \| -0.032 \| % BF \| \| 0.50 \| -0.163 \| -0.296 \| -0.029 \| BMI \| \|  \| -0.142 \| -0.245 \| -0.039 \| % BF \| \| 0.55 \| -0.141 \| -0.292 \| 0.010 \| BMI \| \|  \| -0.145 \| -0.255 \| -0.034 \| % BF \| \| 0.60 \| -0.164 \| -0.338 \| 0.011 \| BMI \| \|  \| -0.130 \| -0.244 \| -0.017 \| % BF \| \| 0.65 \| -0.094 \| -0.266 \| 0.078 \| BMI \| \|  \| -0.140 \| -0.261 \| -0.019 \| % BF \| \| 0.70 \| -0.012 \| -0.238 \| 0.215 \| BMI \| \|  \| -0.082 \| -0.245 \| 0.082 \| % BF \| \| 0.75 \| -0.045 \| -0.277 \| 0.186 \| BMI \| \|  \| -0.030 \| -0.227 \| 0.167 \| % BF \| |  |  |  |  |
| --- | --- | --- | --- | --- | --- | --- | --- | --- | --- | --- | --- | --- | --- | --- | --- | --- | --- | --- | --- | --- | --- | --- | --- | --- | --- | --- | --- | --- | --- | --- | --- | --- | --- | --- | --- | --- | --- | --- | --- | --- | --- | --- | --- | --- | --- | --- | --- | --- | --- | --- | --- | --- | --- | --- | --- | --- | --- | --- | --- | --- | --- | --- | --- | --- | --- | --- | --- | --- | --- | --- | --- | --- | --- | --- | --- | --- | --- | --- | --- | --- | --- | --- | --- | --- | --- | --- | --- | --- | --- | --- | --- | --- | --- | --- | --- | --- | --- | --- | --- | --- | --- | --- | --- | --- | --- | --- | --- | --- | --- | --- | --- | --- | --- | --- | --- | --- | --- | --- | --- |
|  |  |  |  |  |
|  |  |  |  |  |
|  |  |  |  |  |
|  |  |  |  |  |
|  |  |  |  |  |
|  |  |  |  |  |
|  |  |  |  |  |
|  |  |  |  |  |
|  |  |  |  |  |
|  |  |  |  |  |
|  |  |  |  |  |
|  |  |  |  |  |
|  |  |  |  |  |
|  |  |  |  |  |
|  |  |  |  |  |
|  |  |  |  |  |
|  |  |  |  |  |
|  |  |  |  |  |
|  |  |  |  |  |
|  |  |  |  |  |
|  |  |  |  |  |
|  |  |  |  |  |
